# The Effectiveness of Music in Pediatric Healthcare: A Systematic Review of Randomized Controlled Trials

**DOI:** 10.1155/2011/464759

**Published:** 2010-09-30

**Authors:** Karline Treurnicht Naylor, Shauna Kingsnorth, Andrea Lamont, Patricia McKeever, Colin Macarthur

**Affiliations:** ^1^Bloorview Research Institute, Holland Bloorview Kids Rehabilitation Hospital, Toronto, ON, Canada M4G 1R8; ^2^Lawrence S. Bloomberg Faculty of Nursing, University of Toronto, Toronto, ON, Canada M5T 1P8; ^3^Department of Paediatrics, Faculty of Medicine, University of Toronto, Toronto, ON, Canada M5S 1A8; ^4^Department of Music Therapy, Faculty of Music, Wilfrid Laurier University, Waterloo, ON, Canada N2L 3C5

## Abstract

The aim of this study was to systematically review the effectiveness of music on pediatric health-related outcomes. Five electronic databases were searched for randomized controlled/crossover trial designs published between 1984 and 2009. Eligible studies used music as a therapy or intervention, included participants 1 to 18 years, and focused on at least one health-related outcome (with the exclusion of procedural pain). Seventeen studies met the inclusion criteria. Quantitative synthesis was hampered by an inability to aggregate data arising from heterogeneity of interventions, outcomes and measurement tools. Qualitative synthesis revealed significant improvements in one or more health outcomes within four of seven trials involving children with learning and developmental disorders; two of three trials involving children experiencing stressful life events; and four of five trials involving children with acute and/or chronic physical illness. No significant effects were found for two trials involving children with mood disorders and related psychopathology. These findings offer limited qualitative evidence to support the effectiveness of music on health-related outcomes for children and adolescents with clinical diagnoses. Recommendations for establishing a consensus on research priorities and addressing methodological limitations are put forth to support the continued advancement of this popular intervention.

## 1. Introduction

Formally defined, music therapy is the systematic use of music or musical elements—along with the resulting interpersonal relationship with a trained music therapist—to achieve optimal health outcomes for a client or group of clients [1–3]. Musical interventions include passive listening to prerecorded music and active music making [2]. Both types of interventions have been applied in diverse patient populations [2, 4–24]. Long considered a “universal language” that can be perceived early in development [25, 26], the noninvasive, pleasurable, flexible, and dynamic nature of music make it particularly relevant as a treatment medium for children and adolescents [5, 6, 12, 14, 16–18]. 

Six meta-analyses examining the use of music in the context of pediatric healthcare have been published [2, 12, 14, 16–18]. Two of these reviews focused exclusively on the effectiveness of music on reducing procedural pain. Standley and Whipple conducted a meta-analysis of 29 observational studies involving infants, children, and adolescents undergoing invasive and noninvasive medical procedures and concluded that musical interventions reduced pediatric pain, anxiety, and distress [14]. Likewise, Klassen et al. calculated a small to medium effect of music in this context from 19 randomized control trials (RCTs) [18]. These findings are consistent with meta-analyses examining the effects of music on pain, anxiety, and other indicators of stress in hospitalized adults [2, 11, 19–21]. Dileo and Bradt conducted a broad meta-analysis of medical music therapy, combining RCTs and observational studies within 11 medical specialties; they cited a moderate effect within the subspecialty of pediatrics from 11 trials largely related to medical procedures [2].

The three remaining meta-analyses focused on specific populations. Consistent with reviews in adult mental health [7–9, 15], Gold et al. reported a large positive effect of music therapy on objective outcomes including developmental milestones and problem behavior, and a medium positive effect on subjective outcomes including self-concept and social skills for children and adolescents with behavioral, emotional, and/or developmental disorders [12]. A large effect of musical interventions on cognitive skill and social behavior in autistic children was reported by Whipple, based on 9 observational studies identified in a narrow literature review [16]. Neither Whipple [16] nor Gold et al. [12] provide a comprehensive description of steps taken to minimize bias in study selection and data extraction [27, 28]. Using more rigorous methodology and focusing exclusively on RCTS, Gold et al. reported a medium effect of music therapy on nonverbal communication and a small to medium effect on verbal communication in children with autism and related pervasive developmental disorders [17]. 

Although these meta-analytic findings are supportive of the effectiveness of music, the reviews are narrow in focus. For example, of notable absence are children with acquired and/or congenital physical disabilities despite the use of music therapy as a habilitation tool with these populations [23, 24, 29]. To build on the findings of previous papers, we undertook a comprehensive systematic review of randomized controlled trials of music therapy and musical intervention in pediatric healthcare. This paper does not focus on particular clinical populations or specific outcomes, but examines the effectiveness of music on health-related outcomes in children and youth with a variety of clinical conditions in a variety of settings (educational, outpatient, inpatient, and research).

## 2. Methods

A systematic review of the peer-reviewed literature was undertaken following the guidelines outlined in the PRISMA (Preferred Reporting Items for Systematic reviews and Meta-analyses) Statement. This statement includes a 27-item checklist to improve the conduct of systematic reviews and meta-analyses of health care interventions by ensuring transparent and complete reporting [30, 31]. 

### 2.1. Search Strategy

**Figure 1:**
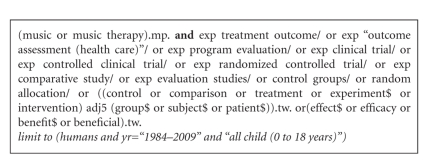
Example of series of keywords and descriptors used to search the Ovid Medline database.

The search strategy and database selection were developed through consultation with a research librarian. The search strategy contained a broad series of subject headings and keywords relating to music or music therapy and outcome-driven research design. Previously published meta-analyses were also reviewed to guide the development of the search strategy and identify pertinent publications [12, 14, 16–18]. The following international electronic databases were searched on the 4th March 2009: Ovid Medline (Medical Literature Analysis and Retrieval System Online), 1950 to February, Week 3, 2009; Embase, 1980–2009, week 9; PsycInfo, 1967 to February, Week 4 2009; AMED (Allied and Complementary Medicine), 1985–February 2009; and CINAHL (Cumulative Index of Nursing and Allied Health Literature), 1983–2008. There were no language restrictions. The search was limited to the time period 1984–2009 inclusive and by age (0–18 years) using filters unique to each database. An example of the search strategy is provided in Figure 1; minor modifications were made as required within individual databases.

### 2.2. Study Selection

Retrieved records were imported into RefWorks and duplicates removed [32]. Non-English abstracts and full-text records were translated. Two reviewers (KTN and SK) independently screened titles and abstracts for relevance; potentially relevant studies were reviewed independently in full by KTN and AL. Studies were included if they met the following 6 criteria: (1) examined the effectiveness of a music intervention, (2) involved a clinical population in a healthcare, research, or education setting, (3) involved children and adolescents between 1 and 18 years of age (or reported a mean age within this range), (4) used a RCT design (parallel or crossover), (5) reported at least one quantifiable outcome measure, and (6) published between 1984 and 2009. 

The focus of this paper was to determine the effectiveness of music as an intervention or therapy, regardless of delivery mode (i.e., by a trained music therapist, health professional, or researcher). Thus, studies examining music education, acoustic or auditory stimulation, or nonmusical sounds (e.g., white noise) were excluded. Given the recent systematic review examining RCTs for procedural pain and anxiety in children [18], trials of the effectiveness of music for children undergoing a medical or dental procedure were also excluded. Ineligible studies were filed with a reason for exclusion, and discrepancies between reviewers were resolved through discussion until consensus was reached. 

### 2.3. Data Extraction

Data from included studies were extracted and compiled by KTN and verified by SK and AL using a standard form.  Table 1 includes information about each study (authorship, year of publication, country, recruitment setting, and experimental design), participants (sample size, gender, population, and age), intervention (treatment, delivery, participant involvement, and dosage), and quality rating.  Table 2 describes outcomes, measurement tools, analyses, and key findings for each study.

### 2.4. Data Analysis

Data quality was assessed (SK and AL) using the PEDro Scale [50]; a comprehensive and reliable measure of the methodological quality of clinical trials [51–53]. This scale assigns a total possible score of 10 based on the following criteria: (1) random allocation, (2) concealed allocation, (3) baseline similarity, (4) blinding of all subjects, (5) blinding of all therapists, (6) blinding of all assessors, (7) participant retention and data collection, (8) intention to treat analysis, (9) between-group statistical analysis, and (10) sufficiency of statistical reporting [50].

Because of heterogeneity in the study populations, interventions used, and outcome measures applied, it was neither feasible nor appropriate to conduct a meta-analysis. Therefore, the findings were synthesized in a qualitative manner. To facilitate this synthesis, the final studies were grouped into four broad categories based on the primary diagnoses or conditions of the study participants. “Learning and developmental disorders” includes children with autistic spectrum disorders, attention deficit-hyperactivity disorder, learning disabilities, and developmental delay. The category “stressful life events” includes children experiencing losses or trauma such as bereavement, divorce, or refugee status. A third category—“mood disorders and related psychopathology”—includes children diagnosed with depression or other psychiatric conditions. The final category “acute and/or chronic physical illness” was reserved for children with physical illnesses or conditions. 

## 3. Results

### 3.1. Study Characteristics

**Figure 2:**
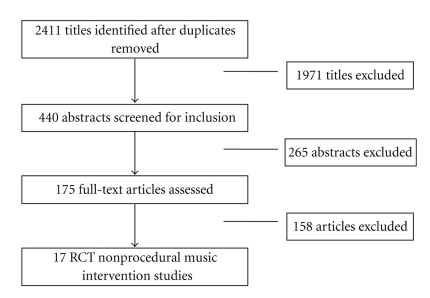
Flow of studies through the systematic review process.

Of the 2411 titles identified, 17 studies met the inclusion criteria [33–49]; Figure 2 describes the flow of studies through the selection process. The final sample comprised 9 parallel (randomization of individuals), 2 cluster parallel (randomization of groups of individuals), and 6 crossover RCTs. 

Selected trials included a total of 575 participants; approximately 50% were male (1 study did not provide data by gender). Sample sizes ranged from 8 to 134 participants with a median trial sample size of 22. With the one exception of a trial involving participants less than 2 years of age [48], the trials focused heavily on elementary school age children [33–36, 41, 42, 46, 47, 49], adolescents [43, 44], or a combination [37–40, 45]. Reflecting the diagnostic range of participants, recruitment settings included the community [37, 49], hospital inpatient units [45–47], outpatient clinics [33, 36, 43, 48], schools [34, 35, 40–42], and residential educational [38, 39] and psychiatric [44] facilities. 

Outcomes included observed behavior and performance [34–36, 38, 39, 43, 46], physiological signal detection [37, 43], documentation of clinical symptoms and related behaviors [33, 36–40, 42, 49], and participants' self-reported perceptions and beliefs [41–45, 47, 48]. Trials employed frequency counts [34–36, 39, 46, 49], validated questionnaires [33, 36–45], and other nonvalidated tools or ratings [36–38, 47, 48] completed by parents, teachers, youth, and/or raters. Although 11 trials involved multiple collection periods [33, 35, 36, 39–41, 43, 44, 46, 48, 49], only 2 trials assessed durability of change beyond the immediate end of treatment; Oelkers-Ax et al. assessed outcomes 8 weeks postintervention [49] and DeLucia-Waack and Gellman, at 3 months postintervention [41].

Methodological quality was poor with an overall median PEDro score of 3 (min = 2, max = 6); classifying the studies, 9 were of low quality (score ≤3) and 8 of moderate quality (4 ≤ score ≤ 6). Although all reported random allocation, two studies allocated at the level of the group (i.e., by school or by counselor) [41, 42], one study reported using sequential assignment tables [46], and two studies did not achieve group equivalency [33, 38]. Failure to provide adequate details of key baseline descriptors [34, 35, 37–39, 44–48] and to undertake appropriate statistical analyses [41, 42] was also noted. Dropout was an issue for five studies with final sample sizes less than 85% of the original number allocated to groups [33, 36, 38, 44, 49]; six studies failed to provide sufficient information to render this determination [34, 37, 39, 41, 43, 46]. Only one study concealed treatment allocation [49], and three studies employed blinding in outcome assessment [33, 35, 46].

### 3.2. Intervention Characteristics

Study objectives varied greatly; music was used to influence cognitive functioning [34, 35], improve social skills and the achievement of other developmental milestones [33, 36], ameliorate coping and affect [40–48], and reduce physical and physiological symptoms [37, 43, 49] and maladaptive behaviors and beliefs [36–43]. To determine effectiveness, musical interventions were compared to no music [33, 37–40, 42, 48], standard clinical practice such as psychoeducation, social work, medical play, and pharmacology [41, 42, 47, 49], or other musical and nonmusical therapies or interventions such as free play, verbal rehearsal, art, or self-relaxation [34–36, 43–46, 48]. 

Seven trials exclusively employed prerecorded music [34, 35, 37, 41, 43, 44, 48]. With the exception of DeLucia-Waack and Gellman [41], these particular interventions involved passive listening by participants, guided by the researcher or health professional. Seven trials employed live music consisting of a range of percussion instruments, songs, and rhythm-based activities and promoted active initiation, improvisation, and music creation by participants [33, 36, 39, 42, 45, 47, 49]. Three trials employed a combination of these presentation modalities [38, 40, 46]. 

Sessions were offered one-to-one with individual participants [33–37, 43–49] or to small groups [37–41]. Within these sessions, there was great variability in treatment “dosage”, ranging from a single session of 15 to 20 minutes in duration [46] to 30-minute sessions twice daily over 12 weeks [48]. Delivery of musical interventions varied but was predominantly provided by music therapists [33, 36, 38–40, 45, 48, 49], other allied health care professionals (e.g., social worker) [41, 47], or a combination [42, 46]. In the remaining studies, a researcher provided the intervention [34, 35, 37, 43, 44].

### 3.3. Qualitative Synthesis: Clinical Diagnosis

#### 3.3.1. Learning and Developmental Disorders

Two trials investigated the influence of music therapy on normative development and cognitive functioning in children with developmental delay [33, 34]. No significant effect of improvisational music therapy on the achievement of developmental communication-related goals was reported in a moderate quality trial by Aldridge et al. [33]. Claussen and Thaut found that exposure to familiar music resulted in a significantly higher recall accuracy of multiplication tables compared with verbal rehearsal in a trial of low quality [34]. 

Two small trials (*N* = 10) of moderate quality examined the effect of music on cognitive functioning and social behavior in children with autism [35, 36]. Buday et al. showed that children exposed to recorded music were more likely to remember and imitate signed and spoken words compared with those given rhythm cues. The difference, however, amounted to an average of only one word [35]. Kim et al. examined improvisational music therapy versus play sessions on joint attention behaviors in autistic boys [36]. A large and significant effect size was found for the Early Social Communication Scales—a structured assessment of individual differences in nonverbal communication skills [54]—driven by positive impacts of music therapy on quality and quantity of eye contact and turn-taking behaviors relative to gesturing and behaviors indicating intent. No significant difference was found using the Pervasive Developmental Disorder Behavior Inventory [55]—a pediatric measure of maladaptive and adaptive behavior [36]. 

Impulsivity and related behavioral outcomes were the focus of three low quality trials involving youth with attention deficit disorders [37–39]. Pratt et al. examined the effect of neurofeedback training with or without prerecorded background classical music on physiological responding, disorder severity, and behavior including impulsivity in children with ADHD or ADD; no significant differences in symptomatology were found [37]. Rickson and Watkins found no differences between music therapy (songwriting, instrumental- and rhythm-based activities) and control groups on a parent and teacher measure of antisocial and disruptive behavior among adolescent boys with varied deficits including ADD and ADHD [38]. In contrast, Rickson examined the effects of instructional versus improvisational music therapy on motor impulsivity among adolescent boys with ADHD and other comorbid disorders. Compared to wait-list controls, both types of music therapy (instructional and improvisational) resulted in a significant increase in accuracy on a motor task and a significant reduction in the teacher-rated Conner's Global Index Restless-Impulsive Scale and the Conner's DSM-IV Hyperactive-Impulsive Scale [56]. No differences were found between the two types of music therapy [39]. 

#### 3.3.2. Stressful Life Events

Coping was the focus of three trials of low to moderate quality involving children who had experienced a major upheaval in their lives [40–42]. Baker and Jones showed that music therapy emphasizing song writing and singing significantly reduced externalizing behaviors, such as aggression, hyperactivity, and conduct issues among newly arrived immigrant and refugee youth as compared to a control group. No significant differences, however, were found for measures of internalizing behaviors, school problems, or adaptive skills [40]. DeLucia-Waack and Gellman reported no significant effects of a song-based music intervention with respect to anxiety, depression, or irrational beliefs compared to traditional psychoeducational approaches in a large trial of 134 children experiencing parental divorce [41]. Positive effects were reported by Hilliard in a comparison of music therapy (songs and instruments) and social work (art and play therapy) approaches to deliver a standardized grief-based curriculum. While both groups experienced a significant decrease in behavioral distress, only the music therapy group experienced a decrease in grief symptoms [42]. 

#### 3.3.3. Mood and Related Psychopathology

Two low quality trials involving adolescents with mood and related affective disorders produced unclear findings [43, 44]. Field et al. examined the effect of popular music on the mood of chronically depressed female adolescents. Relative to a control group, the music group showed a significant decrease in salivary cortisol and EEG activity; however, and of more clinical significance, no differences in observed affective behavior or self-reported mood were found [43]. Similarly, no main effects of popular music (heavy metal versus rock) on self-reported affect using the Positive and Negative Affect Schedule (a measurement of fluctuations in mood) [57] were reported by Wooten [44].

#### 3.3.4. Acute and/or Chronic Physical Illness

Three low to moderate quality trials examined the effects of music therapy on coping among hospitalized children [45–47]; two trials focused primarily on children with cancer [45, 46]. A comparison of the effects of creating visual art and composing electronic music by Colwell et al. found no significant difference between the groups on self-concept from pre- to post-test [45]. In contrast, Robb et al. examined the effect of active music engagement (AME) consisting of songs and instrumental activities on observable coping-related behaviors compared to listening to recorded children's music (ML) or the use of recorded audio storybooks (ASB). Only AME resulted in significant increases in positive facial affect and active engagement; however, both AME and ML led to higher rates of initiation (a measure of a child's environmental exploration and interaction) as compared with ASB [46]. Positive effects were also reported by Froehlich among children with varied diagnoses. Significantly more verbalizations about the experience of being hospitalized were made during music therapy than during play therapy [47]. 

Grasso et al. examined the effects of “treatment” music (specially composed by a music therapist) or familiar children's music compared with no music on child and parent experiences of chest physiotherapy in infants and toddlers with cystic fibrosis. This moderate quality trial found treatment music resulted in a significantly more positive experience for parents and children as compared to familiar music or no music. Neither type of music changed parents' perceptions of time taken to complete therapy [48].

Last, symptomatology was the focus of a trial of moderate quality by Oelkers-Ax et al. comparing the effect of individualized music therapy emphasizing relaxation and techniques for coping with pain, butterbur root extract, or a placebo in combination with education and symptomatic pain treatment on the frequency and severity of migraine headaches. Relative to the placebo, both interventions reduced migraine frequency over an extended period. Music therapy, however, had a more immediate and lasting impact compared to the pharmacological approach, with significant reductions in migraine frequency posttreatment and on follow-up [49].

### 3.4. Qualitative Synthesis: Outcome

#### 3.4.1. Cognitive Functioning

Two trials with low to moderate quality PEDro scores targeted cognitive functioning and reported improvements in recall accuracy of multiplication tables [34], signs, and spoken words [35] following passive exposure to recorded music.

#### 3.4.2. Social Skills and Other Developmental Milestones

Two trials with similar moderate PEDro scores examined the acquisition of social skills and achievement of developmental milestones using standardized assessments of observed behavior following music therapy [33, 36]. No evidence supporting change in social behavior, communication, and other developmental milestones as assessed by the Griffiths Scale [58] and by the Pervasive Developmental Disorder Behavior Inventory [55] is found [33, 36]. A significant improvement in nonverbal communication on the Early Social Communication Scales [54] was reported [36]. 

#### 3.4.3. Coping and Affect

Coping and affect were the focus of nine trials [40–48]. No significant differences in affect using self-report or observed affect and behavior were found in two low quality trials involving passive listening to rock and other types of popular music [43, 44]. In contrast, two music therapy trials with low to moderate PEDro scores yielded positive effects of observed affect, frequency of engagement and initiation [46], and verbalizations [47]. Listening to a specially recorded “treatment” music composition also had a positive effect on parental reports of children's experience of cystic fibrosis chest treatments (PEDro score = 5) [48]. However self-created music compositions did not improve scores on the Piers-Harris Children's Self Concept Scale [59] (PEDro score = 3) [45].

Differential findings were evident for three low to moderate quality trials assessing internalizing and externalizing behaviors using standardized measures [40–42]. Significant effects of song- and instrumental-based activities on externalizing behaviors using the Behavior Assessment System for Children [60] (a teacher report of classroom and playground behaviors) and the Behavior Rating Index for Children [61] (a parental report of behavior problems) were reported for both music therapy trials [40, 42]. Although a significant difference in frequency of internalizing behaviors (e.g., anxiety, depression, and beliefs) was found using the Bereavement Group Questionnaire for Parents [42, 62], no differences were found using the teacher rated Behavior Assessment System for Children [40, 60]. The music intervention (song-based activities) also reported no differences in internalizing behaviors using three self-report measures: the Children's Beliefs about Parental Divorce Scale [63], the Revised Children's Manifest Anxiety Scale [64], and the Children's Depression Inventory [41, 65]. 

#### 3.4.4. Symptomatology

Five trials targeted frequency of symptoms related to clinical diagnoses with varied success [36–39, 49]. In a study with a moderate PEDro score of 6, significant reductions in migraine frequency were reported via a self-report diary following music therapy [49]. Four low to moderate quality studies examined the effects of music on impulsivity and related behavioral symptoms [36–39]. Improvements in accuracy in a tapping task and teacher ratings on Conner's Global Index and DSM-IV subscales [56] were found as a measure of reduced impulsivity following song- and instrumental-based music therapy [39]. In contrast, no changes in disorder severity and related behaviors (maladaptive or antisocial and disruptive) using parental and/or self-report measures were found following exposure to classical music [37] or varied music therapy activities [36, 38]. 

#### 3.4.5. Physiological Measures

Two trials with similar low PEDro scores examined change in affective patterns of EEG responding following passive listening to recorded music [37, 43]. No significant changes were reported by Pratt et al. [37]. In contrast, Field reported significant differences in EEG patterns accompanied by decreased levels of salivary cortisol; the clinical significance of these changes is not clear [43].

### 3.5. Qualitative Synthesis: Other Comparisons

No clear influence of participant involvement (active versus passive) or dosage (length of exposure in minutes) was identified among the treatment effects. Interventions led by a music therapist were more likely to yield significant effects than interventions led by a health professional or researcher. 

## 4. Discussion

### 4.1. Summary of Findings

Over a 25-year period, 17 RCTs examining the effectiveness of music on health-related outcomes in children were identified. While methodological limitations and clinical heterogeneity preclude drawing firm conclusions, a qualitative synthesis of findings suggest some effectiveness of music as an intervention in pediatric healthcare. Reviewing findings as a function of diagnostic category, treatment effects were mixed for children with learning and developmental disorders [33–39]. For example, significant effects were reported for both studies involving children with autism [35, 36], one of two studies involving children with learning or developmental delays [34], and one of three studies involving children with ADD or ADHD [39]. More promising trends were noted for children experiencing stressful life events [40, 41] and for children with acute and/or chronic physical illness [45–49], with positive effects in two of three trials [40, 42], and four of five trials [46–49], respectively. No evidence was found among adolescents with mood disorders and other psychopathology [43, 44].

Turning to clinical outcomes, exposure to music positively affected cognitive functioning and was associated with higher recall accuracy [34, 35]. Clinical symptoms were also improved with significant reductions in migraine frequency [49] and motor impulsivity [39]. As to physiological proxies for clinical outcomes, decreased levels of salivary cortisol were found [43] but changes in affective EEG patterns were inconsistent [37, 43] with one of two studies reporting a significant effect [43]. No other changes were found in direct or proxy measures of the severity of the clinical disorders studied [36–38]. With respect to coping, music had a significant impact with demonstrated increases in coping behaviors [46–48] and reduced frequencies of behavior problems associated with grief and distress [40, 42]. However, the effects of music on internalized symptoms related to coping were unclear [40–42, 45]; only one of three studies reported a significant improvement in grief-related symptoms [42], and there was no significant impact on self-concept [45]. Similarly, the impact of music on clinical affect was also unclear [43, 44, 46], with one of two trials reporting improvements in observed affect [46], but no effects noted when changes in affect were self-reported [43, 44]. Finally, inconsistent findings were reported for social behaviors and developmental achievements [33, 36] with one of two trials reporting significant improvements in nonverbal communication [36].

Although previous papers have explored the influence of intervention characteristics [4, 7, 14, 66] and noted differential effects of participant involvement [14], delivery [14], and type of music [4], no clear trends were discernible in the current review. Consistent with Dileo's finding of greater effects for music therapy [66], significant results were reported more often for trials employing the systematic use of music with a trained music therapist than for trials employing no music therapist. In the absence of a meta-analysis, however, the size of these treatment effects cannot be established. 

### 4.2. Strengths and Limitations

To address the limitations of previous systematic papers [12, 14, 16, 18], we undertook an international search following the strict guidelines of the PRISMA reporting statement [30, 31] and guided by a research librarian. The search was not limited by language, clinical diagnosis, or outcome, and it focused solely on RCTs—the gold standard of experimental design. Quality was ascertained using the PEDro rating system [50] which is well-suited to assess studies evaluating clinical interventions [51–53]. Despite efforts to enhance generalizability through broad definitions of musical interventions and health (which included social, physical, and mental well-being), the final sample comprised a narrow range of diagnostic conditions. 

Within this sample, variation in outcomes and/or outcome measures precluded formal aggregation of the results and completion of a meta-analysis—thereby limiting definitive conclusions of the effectiveness of musical interventions. Despite the systematic design of this paper, the exclusive focus on published trials does raise the risk of a publication bias and overestimation of treatment effects [67]; previous meta-analyses, however, have failed to find such evidence [12, 18]. Of greater concern is the relatively weak methodological quality of the trials included as based on the PEDro scores; the highest score obtained was 6 out of 10 [35, 40, 49]. Very few trials provided detailed accounts of methods of randomization and allocation; significant baseline differences were noted between comparison groups in two trials [33, 38]. Quality of statistical analysis and reporting were also poor; for example, both DeLucia-Waack and Gellman, and Hilliard failed to account for clustering in their statistical analyses [41, 42]. 

The issue of methodological quality has been raised repeatedly in both the pediatric and adult literature around music therapy. It is, however, but one of the issues impeding meta-analytic synthesis of the music literature. Of perhaps equal concern is the lack of standardization of interventions, including both music therapy and musical interventions, and appropriate controls. Further limiting the task is the extensive outcome and measurement heterogeneity within and across diagnostic groups [5, 9, 12, 13, 15, 17, 19–21].

### 4.3. Future Directions

Collectively, these factors restrict the collection of definitive data on the effectiveness of music in pediatric healthcare. The issue is not simply a lack of research but rather a lack of high quality research. As other authors have noted, the field would benefit from a strict adherence to methodological quality [5, 7, 12, 51–53], transparent reporting and use of published guidelines (e.g., CONSORT Statement) [68–72], and consensus on research priorities [73–75]. Priority issues are larger sample sizes, standardized interventions, consistent outcome measures, simpler (parallel-group) designs, and high quality execution. Many of the studies identified involved complex cross-over designs, multiple interventions with multiple outcome measures, and small sample sizes putting them at higher risk for methodological errors and uncertainty around the statistical power of the comparisons. In addition, clinically relevant validated outcome measures are required, and these outcomes need to be measured over time to establish durability of change; none of the studies included long-term follow-up assessments. In the absence of methodological rigor, the literature will remain heterogeneous and the opportunity to define the evidence supporting (or not) the effectiveness of music therapy will be lost.

## 5. Conclusion

This paper is the first systematic review to examine the effectiveness of music among varied pediatric conditions and settings. The findings offer limited qualitative evidence to support the effectiveness of music for children with learning and development disorders and acute and/or chronic physical illness, and children experiencing stressful life events. No evidence to support the effectiveness of music for children with mood disorders and related psychopathology was found. From a health outcomes perspective, music may be used to enhance cognitive abilities [34, 35], facilitate verbal [47] and nonverbal communication [36], and influence physiology [43]. The emotive qualities of music may reduce the effects of trauma and facilitate coping strategies for difficult environments [40, 42, 46–48]. Music may also reduce symptomatology, such as maladaptive behaviors [39, 40, 42] and migraine frequency [49]. Current understanding of the potential benefits of music in pediatric healthcare is compromised, however, by methodological limitations.

## Figures and Tables

**Table 1:** Characteristics of eligible studies.

Study				Participants			Intervention						Dosage		Quality
First Author, Year	Country	Recruitment setting	Study design	N (n males)	Clinical population	Age range (years)	Treatment	Music delivery^*∞*^	Music type^±^	Participant involvement^×^	Delivery format^●^	Intervention format^≈^	No. of sessions (time)	No. of weeks	PEDro score (sum/10)
Learning and developmental disorders															

Aldridge, 1995 [33]	Germany	Private practice clinic	Crossover RCT	8 (2)	Developmental delay	4–6.5	Group 1 (*n* = 5): Music therapy (Nordoff & Robbins adaptation); Group 2 (*n* = 3): Initial non-treatment group; cross-over occurred 3 times	MT	L	A	O	I	24 (30 m)	48	5

Claussen, 1997 [34]	USA	Special education facility	Parallel RCT	21 (12)	Learning disabilities	9–11	Group 1: Familiar music; Group 2: Verbal condition; both groups rehearsed multiplication problems	R	R	P	O	S	1 (<60 m)	<1	3

Buday, 1995 [35]	USA	Public school (special education program)	Crossover RCT	10 (8)	Autism	4–9	Group 1 (*n* = 5): Music condition; Group 2 (*n* = 5): Rhythm condition; 5 trials/day	R	R	P	O	S	8 (n/a)	2	6

Kim, 2008 [36]	Korea	Ambulatory care clinic	Crossover RCT	10 (10)	Autistic disorder	3–5	Group 1 (*n* = 5): Block of improvisational music therapy (instrumental) + block of play (toys); Group 2: Reversed block order	MT	L	A	O	I	12 (30 m)	24	4
Pratt, 1995 [37]	Canada	Community	Parallel RCT	19 (17)	ADD or ADHD	6–18	Neurofeedback sessions with or without background classical music	R	R	P	O	S	39 (<60 m)	13	3

Rickson, 2003 [38]	New Zealand	Special education residential facility	Parallel RCT	15 (15)	Intellectual, social and emotional deficits including ADD/ADHD	11–15	Group 1 (*n* = 4): Waitlist control group; Group 2 (*n* = 6) & Group 3 (*n* = 5): Music therapy sessions (favorite music; instrumental and rhythym based activities).	MT	L & R	A & P	G	S & I	16 (30–45 m)	8	3

Rickson, 2006 [39]	New Zealand	Special education residential facility	Crossover RCT	13 (13)	ADHD and other comorbid disorders	11–16	Group 1 (*n* = 5): Waitlist control group. Group 2 (*n* = 4): 1 block of improvisational + 1 block of instructional music therapy. Group 3 (*n* = 4): reversed order	MT	L	A	G	S & I	16 (30–45 m)	20	2

Stressful life events															

Baker, 2006 [40]	Australia	ESL secondary school	Crossover RCT	31 (11)	Newly arrived immigrant and refugee adolescents	11–16	Groups 1 (*n* = 15): 2 blocks music therapy (instrumental and song based activities) + 2 blocks no music therapy; Group 2 (*n* = 16): reversed order	MT	L & R	A & P	G	S & I	20 (30–40 m)	20	6
DeLucia-Waack, 2007 [41]	USA	Elementary school	Cluster parallel RCT	134 (67)	Children from divorced and/or separated families	5–10	Group 1 (*n* = 60): Music intervention (song based); Group 2 (*n* = 74): Traditional psychoeducation	HP	R	A	G	S	8 (45 m)	8	3

Hilliard, 2007 [42]	USA	Elementary schools	Cluster Parallel RCT	26 (14)	Children experiencing bereavement	5–11	Group 1 (n=8): Orff-based music therapy; Group 2 (*n* = 9): Traditional social work interventions; Group 3 (*n* = 9): waitlist control group	MT & HP	L	A	G	S	8 (60 m)	8	4

Mood disorders and related psychopathology															

Field, 1998 [43]	USA	Ambulatory care clinic	Parallel RCT	28 (0)	Chronic depression	14–19	Group 1 (*n* = 14): Music intervention (Rock music); Group 2 (*n* = 14): Self-relaxation intervention	R	R	P	O	S	1 (23 m)	<1	3

Wooten, 1992 [44]	USA	Inpatient psychiatric facility	Crossover RCT	35 (14)	Psychopathology (affective, behavior, or substance abuse)	12–18	Group 1 (*n* = 17): Music intervention (1980s heavy metal music + 1980s popular music); Group 2 (*n* = 18): reversed order. Both groups began in baseline (reading material)	R	R	P	O	S	2 (20 m)	<1	3

Acute and/or chronic physical illness															

Colwell, 2005 [45]	USA	In-patient	Parallel RCT	24 (15)	Acute or chronic illness (>75% oncology)	7–18	Group 1 (*n* = 12): Art (drawing) composition group; Group 2 (*n* = 12): Music (computerized instrumental) composition group	MT	L	A	O	I	1 (45–60 m)	<1	3
Robb, 2008 [46]	USA	In-patient	Parallel RCT	83 (n/a)	Chronic illness (100% oncology)	4–7	Group 1 (*n* = 27): Active music engagement (AME); Group 2 (*n* = 28): Music listening (ML) and Group 3 (*n* = 28); Audio storybooks (ASB) formed two control groups	MT & HP	L & R	A & P	O	S	1 (15–20 m)	<1	3

Froehlich, 1984 [47]	USA	In-patient	Parallel RCT	39 (22)	Acute or chronic illness	5–12	Group 1 (*n* = 20): Music therapy (instrumental and song based activities); Group 2 (*n* = 19): Medical play therapy (storybook & free play)	HP	L	A	O	S	1 (30 m)	<1	4

Grasso, 2000 [48]	Australia	Ambulatory care clinic	Parallel RCT	21 (10)	Cystic fibrosis	0.38–2	Group 1 (*n* = 10): Unfamiliar instrumental treatment for 2 blocks; Group 2 (*n* = 11): No music (Block 1; control) and familiar children's music (Block 2; placebo). Both groups received routine chest physiotherapy.	MT	R	P	O	S	42–168 (30 m)	12	5
Oelkers-Ax, 2008 [49]	Germany	Community	Parallel RCT	58 (40)	Migraine	Mean = 10	8-week baseline condition; Group 1 (*n* = 19): Butterbur root extract; Group 2 (*n* = 20): Music therapy (adaptation of the Heidleberg model); Group 3 (*n* = 19): Placebo	MT	L	A	O	S	12 (n/a)	28	6

RCT: Randomized controlled trial.

^*∞*^MT: Music therapist; R: Researcher; HP: Health professional.

^±^R: Prerecorded music; L: Live music.

^×^A: Active involvement of participant (e.g., instrumental improvisation, song learning and signing, etc); P: Passive presentation to participant (e.g., listening).

^●^G: Sessions offered to group; O: Sessions offered one-to-one.

^≈^S: Standardized intervention; I: Individualised intervention.

**Table 2:** Outcome measures and results of eligible studies.

Study	Outcome			Findings			
Trial	Measure	Scale	Analysis	Result			*P*-value
Learning and developmental disorders							

				Change from baseline:	Group A (MT) = 7.96	Group B (no MT) = 4.60	.045
Aldridge et al., 1995 [33]	Developmental milestones (locomotor development; personal-social; hearing and speech; hand-eye coordination; performance tests; practical reasoning)	Griffiths Scale	Repeated measures ANOVA				
				Change after crossover:	Group A (no MT) = 3.92	Group B (MT) = 5.83	NS

Claussen and Thaut, 1997 [34]	Recall accuracy of multiplication tables	Test of multiplication problems	ANCOVA	Mean accurate responses (SE):	Pre	Post	
				Music	0.9 (.46)	3.5 (.59)	.0001
				Verbal	1.6 (.48)	2.1 (.62)	

Buday, 1995 [35]	Number of correctly imitated signed and spoken words	Scored by independent observer	ANOVA	Group means (SD) for correctly imitated words:	Music	Rhythm	
				Sign	5.10 (2.89)	4.00 (2.83)	<.05
				Speech	4.20 (3.36)	3.20 (2.94)	<.02

Kim et al., 2008 [36]	Joint attention skills and pro-social behaviors; nonverbal social communication skills	Pervasive Developmental Disorder Behaviour Inventory-C (PDDBI); Early Social Communication Scales (ESCS); Video analysis	Repeated measures ANOVA	Effect size (95% CI) for music therapy vs. play session:			
				PDDBI: 0.79 (−0.14 to +1.71)			NS
				ESCS: 0.97 (+0.20 to +1.74)			<.05
				Duration of behaviors:			
				Eye contact: MT > Play			<.0001
				Turn taking: MT > Play			<.0001

Pratt et al., 1995 [37]	EEG frequency band activity; severity of ADD/ADHD; adaptive and maladaptive behaviours	EEG signal (A620 Assessment Software); McCarney Test (parents); Likert scale (parents)	Wilcoxon signed-rank test	Pre-post change in EEG power for ADD children$\hat{\;\;\;\;}$:			
				Band Activity	Music	No music	
				Beta band	−1.54	−1.29	NS
				Alpha band	−2.74	−3.49	NS
				Theta band	−3.66	−0.73	NS
				McCarney Test			NS
				Likert Ratings			NS

Rickson and Watkins, 2003 [38]	Aggressive behaviours: disruptive and antisocial	Developmental Behaviour Checklist (DBC); Video analysis	Repeated measures ANOVA	Mean change for DBC Subscale^⋆^:			
					Teacher	Parent	
				Group 1 (Control)	−1.00	−6.00	
				Group 2 (MT)	−1.80	−1.80	NS
				Group 3 (MTl)	+2.83	+1.00	NS
				Positive & negative events:			
				Group 1 vs Group 2 vs Group 3			NS
Rickson, 2006 [39]	Motor impulsivity	Synchronised tapping task (STT); Conners' Rating Scales (teacher rated)	Unpaired *t*-tests and multi-way ANOVA	Group (Mean # of errors):	Pre	Post	
				(1) NO music	20.13	22.43	
				(2) AB (Improv/Instruct)	20.91	11.56	.02
				(3) BA (Instruct/Improv)	20.89	12.18	.02
				Conner's DSM IV Total:			
				Group 1 > (Group 2 + Group 3)			.02
				Conner's Global Index Scale:			
				Group 1 > (Group 2 + Group 3)			.03

Stressful life events							

Baker and Jones, 2006 [40]	Classroom behaviours: externalising, internalising, school, Behavioral Symptom Index (BSI), adaptive skills	Teachers completed Behaviour Assessment System for Children (BASC)	MANCOVA	F-statistic (df = 21) for Treatment (music/no music) × time:	Externalising: 2.21		.01
					Internalising: 0.32		NS
					Behavior Symptom Index: 2.57		.07
					School problems: 0.89		NS
					Adaptive skills: 0.53		NS

DeLucia-Waack and Gellman, 2007 [41]	Beliefs about divorce and affective measures	Revised Children's Manifest Anxiety Scale (RCMAS); Children's Depression Inventory (CDI); Children's Beliefs about Parental Divorce Scale (CBPDS)	MANOVA	Treatment (music therapy/ psychoeducation) × time:	Anxiety Depression Irrational Beliefs	F (6,127) = 0.487	NS
						F (10,123) = 1.416	NS
						F (12,111) = 0.988	NS

Hilliard, 2007 [42]	Grief symptoms and behavioural distress	Behaviour Rating Index for Children (BRIC); Bereavement Group Questionnaire for Parents (BP)	Within-group Wilcoxon signed-ranks tests	Change in BRIC:	Control		NS
					Music therapy		.01
					Social work		.04
				Change in BP:	Control		NS
					Music therapy		.01
					Social work		NS

Mood disorders and related psychopathology							

Field et al., 1998 [43]	Behaviour; mood; stress; left frontal activation	1) Behaviour Observation Scale (BOS); (2) Depression Adjective Checklist (DACL); (3) Salivary Cortisol; (4)EEG recording	Repeated measures MANOVA and post hoc tests	Mean scores for music group (control group):			
				Before	During	After	
				(1) 14.0 (15.1)	14.2 (14.9)	14.7 (14.8)	NS
				(2) 9.5 (8.9)	— (–)	9.7 (9.1)	NS
				(3) 1.3 (1.5)	— (–)	0.5 (1.3)	.02
				(4) − .15(−.13)	−.07(−.11)	−.08(−.11)	.05
Wooten, 1992 [44]	Fluctuations in mood	Positive and Negative Affect Scales (PANAS)	Repeated measures ANOVA	Negative affect	Treatment (none, rock or heavy metal) × time: F (2, 204) = 0.25,		NS
				Positive affect	Treatment (none, rock or heavy metal) × time: F (2, 204) = 1.28		NS

Acute and/or chronic physical illness							

Colwell et al., 2005 [45]	Self-concept	Piers-Harris Children's Self Concept Scale (PHCSS)∗	MANCOVA	Pre- to post-test mean differences:	Music composition 2.08	Art composition 2.00	NS

Robb et al., 2008 [46]	Frequency of coping related behaviors	Time sampling of observed behaviours:	Repeated measures ANOVA and post hoc tests	AME	ML	ASB	
		Positive facial affect		18.63 (13.0)	7.7 (7.5)	2.0 (2.3)	AME>ML, ASB; <.0001
		Active engagement		26.03 (4.1)	15.65 (6.2)	15.17 (4.9)	AME>ML, ASB; <.0001
		Active initiation		14.19 (8.3)	15.89 (11.2)	7.43 (6.6)	AME, ML>ASB; <.05

Froehlich, 1984 [47]	Verbalization of hospital experiences	Standardized questionnaires and coding system to rate quality of responses	Chi-Square	% of responses coded:	Music therapy	Play therapy	
				Answer	90%	62%	<.10
				No answer	10%	38%	

Grasso et al., 2000 [48]	Enjoyment and perception of time	7-point bipolar Likert-type Child* & Parent Enjoyment scale (proxy) and Caregiver Perception of Time survey	Kruskal-Wallis	Median (range) group change for Child enjoyment:			
				Treatment music (TM)	Familiar music (FM)	No music (NM)	
				+1.25 (−1.0 to +4.0)	+0.75 (−3.5 to +3.0)	−0.5 (−4.0 to +2.0)	TM vs NM;.03
							FM vs NM; NS
Oelkers-Ax et al., 2008 [49]	Relative reduction in headache frequency	Child-adapted daily headache diary		% reduction from baseline°:	Butterbur, Placebo, Music		
			Repeated measures	Post-test	36.1 ± 57.3, 28.8 ± 39.5, 65.7 ± 31.0		M>P;.005 (at T1) and .018 (at T2)
			ANOVA	Follow-up	58.7 ± 34.6, 31.4 ± 41.8, 63.2 ± 33.9		B>P; NS (at T1) and .044 (at T2)

$\hat{\;\;\;\;}$No significant effects reported for ADHD children.

^⋆^Similar findings for Antisocial Subscale.

*Only child-related outcome measure reported on.

°Only data for treatment completers reported on.
